# Assessment of Risk Factors Associated with Malaria Transmission in Tubu Village, Northern Botswana

**DOI:** 10.1155/2014/403069

**Published:** 2014-03-16

**Authors:** Elijah Chirebvu, Moses John Chimbari, Barbara Ntombi Ngwenya

**Affiliations:** ^1^Okavango Research Institute, University of Botswana, Private Bag 285, Maun, Botswana; ^2^College of Health Sciences, University of Kwazulu-Natal, Howard Campus, Durban 4000, South Africa

## Abstract

This study investigated potential risk factors associated with malaria transmission in Tubu village, Okavango subdistrict, a malaria endemic area in northern Botswana. Data was derived from a census questionnaire survey, participatory rural appraisal workshop, field observations, and mosquito surveys. History of malaria episodes was associated with several factors: household income (*P* < 0.05), late outdoor activities (OR = 7.016; CI = 1.786–27.559), time spent outdoors (*P* = 0.051), travel outside study area (OR = 2.70; CI = 1.004–7.260), nonpossession of insecticide treated nets (OR = 0.892; CI = 0.797–0.998), hut/house structure (OR = 11.781; CI = 3.868–35.885), and homestead location from water bodies (*P* < 0.05). No associations were established between history of malaria episodes and the following factors: being a farmer (*P* > 0.05) and number of nets possessed (*P* > 0.05). Eave size was not associated with mosquito bites (*P* > 0.05), frequency of mosquito bites (*P* > 0.05), and time of mosquito bites (*P* > 0.05). Possession of nets was very high (94.7%). Close proximity of a health facility and low vegetation cover were added advantages. Some of the identified risk factors are important for developing effective control and elimination strategies involving the community, with limited resources.

## 1. Introduction

Understanding how and why vector-borne diseases like malaria remain a persistent problem despite being tools for diagnosis and treatment is essential for developing effective control measures for sub-Saharan Africa. Temperature is one of the key climatic variables that determine the range of malaria transmission and hence global warming is likely to result in an increase in malaria prone areas especially where temperatures have generally been lower than the optimal range of 25–27°C for mosquito development [1]. Furthermore degradation of the environment and social and economic pressures due to population growth may boost the expansion of malaria prone areas [2]. Population migrations, drug and pesticide resistance, and deterioration of health service delivery systems will also influence the level of malaria transmission [3].

The epidemiology of malaria is very complex, involving factors pertaining to the malaria parasites, the insect vectors, the human hosts, and the environment [4]. An understanding of the link between malaria transmission, climatic variables, and other human related factors is therefore necessary for developing appropriate measures that will significantly reduce transmission and perhaps eliminate malaria in endemic areas. In most cases these human related risk factors are known to aggravate the extent of climate related problems.

The level of risk to human populations living in malaria endemic areas varies markedly across continents and also within countries and different areas within the same countries. Several studies have shown that malaria vector distribution, transmission rates, and incidence can vary widely over short distances, between neighbouring villages and even within a single settlement, as a result of small area variations in risk factors [5, 6]. Identification and understanding of this variation are important in the detection of high risk groups and for selective targeting of intervention [7].

Many studies have attempted to identify household and individual level factors associated with malaria. Some of the factors studied include access to health facilities [8], type of housing that people live in [9], proximity of human settlements to vector breeding sites [10–12], vector abundance, socioeconomic status [13], gender, occupation, residential mobility, travel [14], presence of domestic animals near homesteads [15], and use of preventive methods such as bed nets [8]. Information on how these factors interact to expose communities and individuals to malaria infections needs to be investigated systematically in each geographical setting, in conjunction with climate related factors.

Documented information about individual and household risk factors associated with malaria transmission is lacking in Botswana. In order to address this paucity of information we assessed household and individual risk factors that may be contributing to malaria transmission in Tubu village community in the Okavango subdistrict in northern Botswana. The study was conducted in the context of a larger project, Botswana Ecohealth Project (BEP), which is investigating the impacts of hydroclimate change on population health. Apart from contributing towards the objectives of BEP, the results of this study are relevant to an initiative by the Ministry of Health in Botswana to eliminate malaria by year 2016.

## 2. Materials and Methods

### 2.1. Study Area

The study was conducted in Tubu village on the banks of the Thaoge River, one of the distributaries of the Okavango Delta. Tubu village is located in the Okavango subdistrict at an altitude of about 950 m above sea level and between latitude 19°35′S and longitude 22°27′E. According to the national Central Statistics Office report of 2011 there were approximately 71 households with a total population of 483. The major sources of livelihoods were crop and livestock production. The area is subjected to annual flooding but the extent of flooding varies from year to year. The community practices flood recession farming locally known as “*molapo*” farming. They plough their fields along the banks of the river as the flood recedes and in some instances they plant crops taking advantage of a raised water table caused by flooding.

The village has a chief and tribal administration offices. Community development and everyday governance issues pertaining to law and order in the area are conducted at a central place (locally known as the “kgotla”) within the village. The chief is the head of the “kgotla”. Tubu has a Village Development Committee (VDC) established as a local government structure to guide development planning and implementation in the village. The village also has a primary school, a resident social worker, and a health post staffed with a nurse, nurse aid, and a health education assistant. Gumare Hospital, located 10 km east of the village, offers an array of general health services to the local community. The hospital also serves as the district health centre for critically ill patients referred from all district health posts including Tubu.

Malaria cases in the Okavango subdistrict have been fluctuating since 2005 with unconfirmed cases ranging between 4,686 in 2005 and 10,993 in 2006 and confirmed cases ranging between five (in 2012) and 791 (in 2006) (Ministry of Health annual report, 2012). The maximum number of deaths between 2005 and 2012 was 16, recorded in 2006. Reports from Tubu Clinic Register indicated that there were 105 unconfirmed cases in 2006. Data from clinic records for the period of 2005 to 2010 indicated that 2009 had the highest with 131 unconfirmed cases and 2005 the lowest with 50 cases. However during the same period information on Rapid Diagnostic Tests (RDTs) was inconsistent and available only for years 2006, 2008, and 2010 with 9, 4, and 5 cases, respectively.

### 2.2. Study Design and Methods

The study involved descriptive census questionnaire and participatory rural appraisal (PRA) surveys. The surveys were conducted in June 2012. Pretesting of the questionnaire involved trained field research assistants with higher secondary education qualifications and field supervisors with degree qualifications who eventually administered the final questionnaire. Mosquito surveys were conducted for three months prior to the questionnaire and PRA surveys.

#### 2.2.1. Questionnaire Structure

The questionnaire had four thematic sections; first part focused on sociodemographic characteristics such as gender, age, marital status, education level, farming practice, household income, ethnicity, respondent's relationship to household head, employment status, and occupation and the second part included aspects on how the community or individuals got exposed to mosquito bites due to late night activities, location of homesteads in relation to animal shelters and mosquito breeding sites, visits made to other areas outside Tubu village in the last 8 months, and history of malaria episodes in the last 8 months. A short period of 8 months was chosen so as to minimise on-recall bias as the time frame included the previous malaria transmission season which fell between the months of October 2011 and June 2012. The third part of the questionnaire focused on malaria prevention methods being practiced and health delivery services for the study area. The last part involved general observations made at each homestead during the interview with regard to house structure and use, type of eaves, and vegetation cover surrounding the homesteads.

#### 2.2.2. Pretest

To ensure accuracy and good quality data, pretesting of questionnaires was carried out two weeks prior to the actual data collection exercise in Etsha 1, a village with similar environmental and sociodemographic patterns to Tubu village. Nine questionnaires were administered during the pretest survey involving a random selection of participants. Following the pretest survey the questionnaire was revised, on the basis of responses given, for improvement of clarity and addition of essential questions that had been omitted in the original questionnaire. Vague questions in the original questionnaire were either omitted from the final questionnaire or improved. The pretest exercise was also used to standardize the manner in which the interviewers would conduct the interviews and to ensure that they had a common understanding of each question.

#### 2.2.3. Selection of Survey Participants

From the household listing compiled for the BEP project the target sample size, as determined by Raosoft Inc. (2004) sample size calculator, was 61 household heads. However, due to the fact that Tubu is a relatively small village a census survey was done consisting of individuals representing all the 71 households. The questionnaire was administered at the household level to any person who is 18 years and above with preference being given to the heads of the household if they were present. Only one person per household was interviewed. During the interviews the respondents answered some of the questions pertaining to children and other members of the same household such as visits outside study area and possession and use of nets.

#### 2.2.4. Selection of PRA Participants

Qualitative data was derived from a one-day PRA workshop. Random selection of participants for the PRA exercise was done. Numbers 1 to 4 were assigned to individuals from the community who had come to attend the PRAs for the major BEP project. All individuals assigned a similar number were grouped together to participate in the malaria risk survey. PRA technique, using the closed scoring/ranking approach, was used to cross-check on some of the responses from the questionnaire interviews. The maximum number of individuals at any one given time during the PRA discussions was 16, thus providing a total ranking capacity (*n*) of 160.

#### 2.2.5. Malaria Case History

The respondents were asked whether they had suffered from malaria within the past 8 months (the period between October 2011 and June 2012), which included the traditional peak malaria transmission season in Botswana.

#### 2.2.6. Malaria Entomology Survey

Mosquito sampling was conducted in houses, in pit traps, and in larval breeding sites located within the study area.* An. gambiae *s.l. adult mosquitoes were sampled from pyrethrum space spray knockdowns in 6 selected huts used as bedrooms and from 4 pit traps. Larvae were collected from 8 breeding sites and reared to adults. The physiological conditions of the adult mosquitoes collected from indoors and pit traps were scored as non-blood-fed, blood-fed, half gravid, and full gravid. All adult mosquito specimens, from both adult and larval sampling, were identified to species level by the polymerase chain reaction (PCR) technique [16]. A Geographic Positioning System instrument (GPS) was used to determine straight line distances of study homesteads from the nearest mosquito breeding habitats. The different types of mosquito breeding habitats were noted.

### 2.3. Ethical Considerations

Permission to carry out the study was obtained from the Ministry of Health, Republic of Botswana (Permit Number: PPME 13/18/1 Vol. VII (179)). Meetings with the community leadership and other interested community members were held to introduce the study, clearly explaining its objectives. On the day of the interview verbal consent was sought from each participant and the survey objectives were also clearly explained. Respect for privacy was maintained during adult mosquito sampling in selected rooms used as bedrooms. Participants were informed that they could refuse to participate in the study and not be prejudiced. Respondents were assured that all the information captured on the data sheets would be treated with confidentiality and that no answers would be linked to individuals in the final analysis and presentation of findings.

### 2.4. Statistical Analysis

Statistical analysis was carried out using SPSS, version 21.0. However graphs were prepared in MS-Excel 2007. Descriptive statistics were carried out to determine relative frequencies of all the survey variables. Some of the variables had provisions for multiple responses. Appropriate graphs and tables were generated to show differences in the relative frequencies of various variables. Levels of association between various variables were determined by the Pearson *χ* ^2^ test and Fisher's exact test in situations where the expected frequencies were less than five. Where appropriate, *P* values and confidence intervals (CI) for odds ratios (OR) are shown. The level of association between various factors and history of malaria was calculated based on the respondents' views.

## 3. Results

### 3.1. Sociodemographic Characteristics

The maximum number of people residing in a single homestead was 20 and the minimum was one. A total of 483 individuals resided in all the 71 study homesteads, thus giving an average household size of seven people. Farmers comprised 85.9% (61) of the respondents (Table 1). About two-thirds of the respondents had some years of schooling (Table 1).

**Figure 1:**
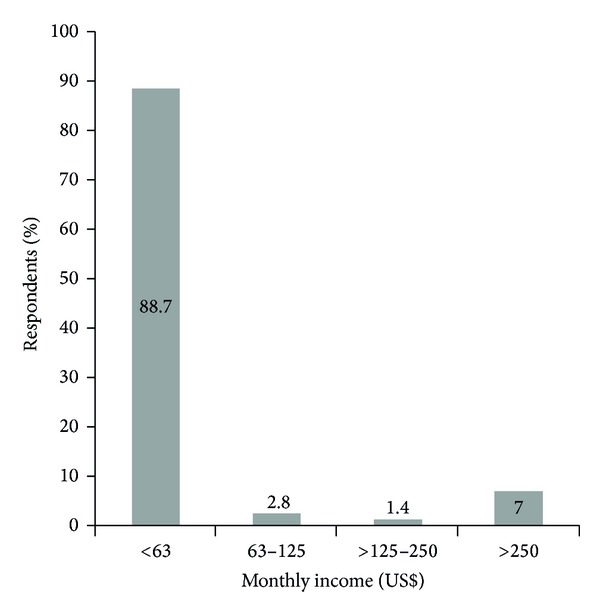
Respondents' monthly household income levels in Tubu village. The exchange rate was US$1 to BWP7.936.

The majority of households earned an income of less than US$63 per month (Figure 1). Only 7% of the respondents earned more than US$250 per month.

### 3.2. Health Facility

Eighty-one point seven percent (58) of the respondents resided within 1 km from the nearest health facility. Eighteen-point three percent (13) resided 1 km to 2 km from the clinic. All the 71 respondents indicated that it took them less than an hour to get service at the clinic from the time they joined the queue.

### 3.3. History of Malaria Episodes

At the time of the study (June 2012) 53.5% (38) of the respondents said that they had suffered from malaria within the past 8 months whilst 46.5% (33) did not remember ever suffering from malaria in their entire life. Forty-seven point nine percent (34) mentioned that at least one member of their family had suffered from malaria within the past 8 months. Among family members who had suffered from malaria, 73.5% (25) were above five years old and 26.5% were below the age of five years. There was an association between household income and history of a malaria episode (Table 2). Almost all of those who had experienced a malaria attack were in the lowest bracket for household monthly income.

### 3.4. House Structure and Vegetation Cover

In the whole village only two house roof types were observed. These were either of grass or iron sheet. A general observation noted that houses in the village were either grass thatched, built of reeds, pole, and mud or home-made bricks (referred to as traditional hut/house) or built of bricks and roofed with iron sheets (referred to as modern hut/house). Among those structures used as bedrooms by the respondents, 52.1% (37) were traditional and 47.9% (34) were modern houses/huts. In traditional houses/huts large eave openings were observed in 89.2% (33) and the rest (10.8% (4)) had small eave openings. In modern houses 2.9% (1) had large eave openings and the rest (97.1% (33)) had virtually no eave openings. There was an association between history of malaria episode and use of traditional huts/houses as bedrooms (Table 3). Majority of individuals who experienced a malaria attack used traditional houses/huts as bedrooms.

Low vegetation cover surrounding homesteads was observed at 81.7% (58) of the homesteads. Moderate vegetation cover was observed at 14.1% (10) of the homesteads whilst only 4.2% (3) of the homesteads were surrounded by dense vegetation cover.

### 3.5. Exposure to Mosquito Bites

Individual contact with mosquito bites was categorized into “always,” “sometimes,” “often,” and “rarely.” Out of 98.6% (70) who experienced mosquito bites, 54.9% (39) mentioned that they “always” got bitten by mosquitoes whilst 28.2% (20) indicated that they “sometimes” experienced mosquito bites. Seven percent (5) and 8.5% (6) “often” and “rarely” got bitten by mosquitoes, respectively. No association was found between house eave size and getting bitten by mosquitoes and between house eave size and frequency of bites (Table 2). Out of the 70 individuals who experienced mosquito bites 84.5% (60) indicated that they received the bites in the evening. Nine point nine percent (7) received bites before dusk. The rest got mosquito bites during daytime (1.4% (1)), after dawn (1.4% (1)), or throughout the day (1.4% (1)). No association was found between house eave size and time of mosquito bites (Table 2).

About 93% (66) of respondents reported that they opened doors in the morning whilst 7% (5) during the day. Ninety-seven point two percent (69) reported that they closed doors in the evening whilst only 1.4% (1) indicated that they closed doors after dawn and 1.4% (1) before dusk. Eighty-eight point seven percent (63) mentioned that they were involved in late outdoor activities that included social gatherings (28.2% (20)), relaxing outdoors (74.6% (53)), preparing meals (50.7% (36)), and doing other things (8.5% (6)). A strong relationship was established between late outdoor activities and history of malaria episode (Table 3). Most of the outdoor activities were said to be done between 2000 hrs and 2200 hrs by 63.4% (45) of the respondents. Only 29.6% (21) indicated that they went indoors before 2000 hrs. About 7% (5) indicated that they stayed outdoors after 2200 hrs. A borderline association between the period spent outdoors and history of malaria episodes was established (Table 2). Ninety-three percent (93% (66)( of the respondents mentioned that they prepared meals outdoors using firewood. Only 2.8% (2) said they used gas for cooking and the remainder (4.2% (3)) used both gas and firewood.

Eighty-five point nine percent (61) of the respondents mentioned that they were fulltime farmers. Among them 60.7% (37) had field structures erected on the farm and these were in the form of mud houses (28.2% (20)), brick houses (2.8% (2)), or other form of materials (21.1% (15)) such as the grass-made temporary shelters locally known as the “mathibelo.” There was no evidence of association between history of malaria episode and being a full time farmer (Table 2).

Seventy-six point one percent (54) of the respondents indicated that they had other residential homesteads elsewhere outside Tubu village. Seventy-three point two percent (52) had other homesteads in Gumare village, 1.4% (1) in the cattle post areas, and another 1.4% (1) in Shakawe village, about 150 km from Tubu village. The term cattle post is widely used in Botswana. It is defined as an area designated solely for keeping domestic animals, mainly cattle, goats, and a few donkeys and horses. Housing structure in a cattle post is mostly of the traditional type. Cattle posts are usually located away from defined residential areas. Majority (69.1% (49)) of respondents owned between 1 and 100 cattle (Table 4). Most of the people who owned cattle kept them at the cattle posts. However cattle roamed freely within the village.

Fifty-nine point two percent (42) of respondents mentioned that they had travelled outside Tubu village in the last 8 months. Ninety-five point two percent (40) of them had travelled to malaria endemic areas in the north of the village whilst 4.8% (2) had travelled to non-malaria-endemic areas. There was a significant association between travel outside Tubu village and history of malaria disease (Table 3). Travelling outside Tubu village could have also exposed individuals to malaria infections. Twenty-six point eight percent (19) of respondents mentioned that 8 months prior to the date of the interview they had received visitors who stayed overnight. They reported that 94.7% (18) of the visitors were coming from malaria endemic areas.

### 3.6. Malaria Prevention Methods

Ninety-four point four percent (67) of the respondents indicated that they owned mosquito nets (insecticide treated or untreated mosquito nets). More than half of the respondents who possessed mosquito nets had not experienced any malaria attack indicating an association between previous malaria episode and possession of mosquito nets (Table 3). Number of nets per household were reported to range from zero (2.8% (2)) to more than six (5.6% (4)), with 39.4% (28) possessing one or two nets, 33.8% (24) possessing three or four nets, and 18.3% (13) possessing five or six nets. However, there was no association between history of malaria attack and the number of mosquito nets owned by a household (Table 2). Insecticide treated mosquito nets (ITNs) were possessed by 78.9% (56) of the responses whilst 50.7% (36) also possessed untreated mosquito nets. It was not unusual to find some households with a mixture of insecticide treated and untreated mosquito nets at the same time. Two point eight percent (2) of the respondents were not sure of the status (treated or not treated) of their mosquito nets indicating poor knowledge on mosquito nets. Ninety-one point five percent (65) of the respondents indicated that they always used mosquito nets. Only 1.4% (1) mentioned that they used mosquito nets more often whilst 5.6% (4) sometimes used them and 1.4% (1) never used mosquito nets. Usage of mosquito nets, among those who always used them, was reported to be relatively high (46.2% (30)) during summer and very low (3.1% (2)) in winter. However night visits to ascertain reported net usage was not done during the study.

**Figure 2:**
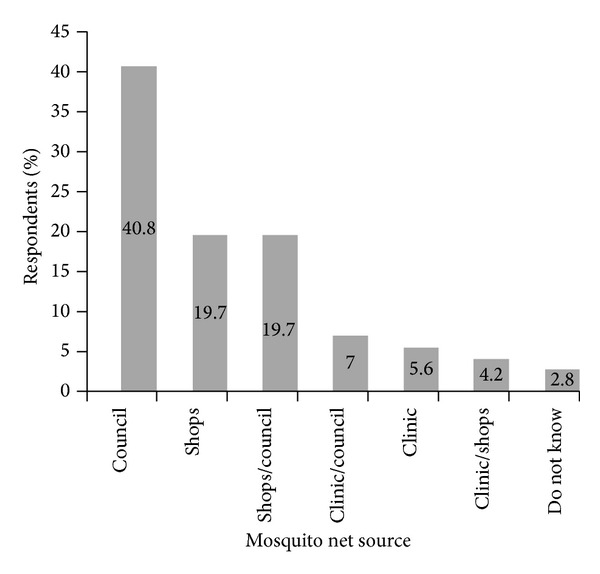
Different sources of mosquito nets possessed by respondents in Tubu village.

Most of the respondents were aware of government's efforts to control malaria through indoor residual spraying (IRS) (97.2% (69)) and distribution of ITNs [97.2% (69)]. Only 2.8% (2) of the respondents were not aware of what the government did to control malaria in Tubu village. Twenty nine respondents got their mosquito nets from the town council (Figure 2). Other respondents acquired their mosquito nets from the shops (14), both shops and town council (14), both clinic and council (5), clinic (4), and both clinic and shops (3). Two of the respondents were not sure about the source of their mosquito nets.

Other personal malaria prevention methods reported as commonly practiced by the community included taking antimalarial drugs (1.4% (1)), clearing of surrounding vegetation (12.7% (9)), avoiding stagnant water (12.7% (9)), wearing long sleeved clothes (2.8% (2)), and use of aerosols (2.8% (2)) and repellents [8.5% (6)]. Only 1.4% (1) said they did nothing to prevent contracting malaria disease.

### 3.7. Participatory Rural Appraisals

A total of 13 individuals (4 males and 9 females) participated in the PRA discussions. During the discussions it was noted that not everyone slept under mosquito nets because of their shortage. This shortage resulted in some household members using emptied maize meal bags to construct “mosquito nets.” However these self-made nets had disadvantages in that they did not kill mosquitoes since they were not treated with an insecticide. Furthermore they were deemed uncomfortable as they tended to retain heat and could not fit well around modern beds. In winter (June-July), most households did not use the nets so often since mosquitoes were not a major problem during that time. The group was satisfied with the spraying exercise but expressed concern regarding coverage of areas inaccessible to the spraying teams due to flooding and also the fact that some households refused to have their homes sprayed claiming that the insecticide caused allergic reactions.

### 3.8. Mosquito Sampling

A total of 86* An. gambiae* s.l. mosquitoes were obtained from larval breeding (22) and adult sampling (64). Using the PCR species identification technique all the 86 specimens were identified as* An. arabiensis,* the principal malaria vector in southern Africa. In addition other nonvector mosquitoes, namely, two* An*,* demeilloni* and one* An. marshallii,* were also identified morphologically. Apart from* Anopheles* species, more than 200 Culicine mosquitoes were also caught resting either indoors or outdoors and breeding in the sites where the vector mosquitoes were found. Forty-eight point four percent (31/64) of the* Anopheles arabiensis* mosquitoes caught resting indoors in selected huts were blood-fed and at different stages of their gonotrophic cycle. Mosquitoes were also observed resting outdoors in animal burrow pits located within the village and close to breeding sites.

**Figure 3:**
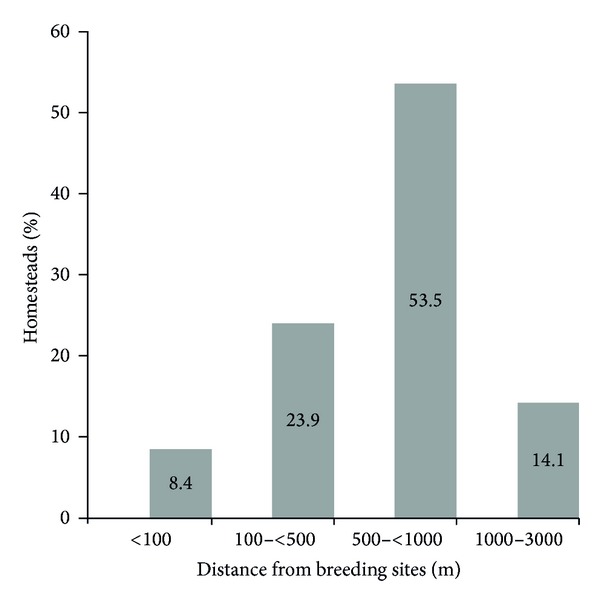
Proportion of respondents' homesteads located at various distances from mosquito breeding sites in Tubu village.

*Anopheles* species larvae were sampled from a variety of breeding habitats. These included vegetated areas at periphery of water bodies, animal hoof prints, open sunlit puddles created by receding floods, and man-made temporary puddles within farming fields. Six homesteads were located within 100 m from mosquito breeding habitats, 17 were between 100 m and 500 m, 38 were between 500 m and 1000 m, and 10 were between 1000 m and 3000 m (Figure 3). Previous malaria episode was significantly associated with distance from water bodies (Table 2).

## 4. Discussion

Majority of our study population earned income far less than the Botswana national poverty datum line pegged at US$81.94 (BWP611.30) by the Ministry of Finance and Development Planning as of 22 April 2013. This is consistent with the country statistics that show that about 44.8% of rural people (30.6% in towns) in Botswana are classified as poor [17]. Such communities have been found to be at greater risk of malaria disease because they are poorer and face higher transmission rates than their urban counterparts [18]. Malaria parasiteamia was associated with a reduced household socioeconomic status in a rural area in Tanzania [13]. Similar associations were observed in the Gambia [19] and Cote d'Ivoire [20]. Social and economic factors aggravate the contribution of climate related factors in influencing the malaria burden. For many social and economic reasons the community in Tubu village never moves out of a large dangerous flood such as that experienced in 2011/2012. According to the World Health Organisation such sedentary communities living in malaria prone areas and who cannot afford to move out of flood-affected areas due to low economic status have an increased chance of acquiring infection [21]. This is particularly true for children, pregnant women, and older people, who form the high risk groups [21].

Other important factors that contributed to individual exposure to mosquito bites were the limited use of personal protection methods such as taking antimalaria tablets and use of aerosols and repellents. Cost of these prevention tools may have been the limiting factor especially in a community regarded as leaving below the national poverty datum line. Similar observations were made in Nouma, Burkina Faso [22], where the use of aerosols was not a popular method perhaps because of the costs associated with such measures. Minimum use of these simple measures not only affect individual exposure to mosquito bites but is also an indicator of very low socioeconomic status, which in itself has been proposed as an important factor associated with malaria [23].

Residential house status or structure has some implications in malaria transmission as poorly constructed houses expose individuals to mosquitoes that may find the dwelling more comfortable as a resting place. Traditional huts/houses with large eave gaps present in Tubu village played a role in allowing free movement in and out of the huts/houses and providing suitable shelter for mosquitoes that eventually attacked the bedroom occupants. Previous studies have shown that traditional grass thatched houses with open eaves and lacking ceilings provided more favourable resting places for mosquitoes and put the occupants at risk of contracting malaria than houses with closed eaves, iron corrugated/asbestos covered roofs, and having ceilings [24]. Nevertheless, in poorly constructed houses where cooking and sleeping take place in the same room, smoke could repel mosquitoes through the eaves at the time that cooking is taking place [9, 25]. However the community in Tubu village did not benefit from the protective effects of smoke since most of the respondents prepared their meals outdoors. Our findings indicated that the community was equally exposed to mosquito bites anytime of the night regardless of the size of eaves. Therefore closing doors early in the evening did not provide any protection since mosquitoes could still freely enter the dwellings through the eaves as was evident from the* Anopheles* and Culicinemosquitoes caught resting indoors.

The presence of endophilic blood-fed* An. arabiensis* mosquitoes in the village verified that the community was at risk of being bitten by an infected vector mosquito. The fact that some study participants spent time in other homesteads away from Tubu village did not make any difference since the structure of the dwellings there was the same and most of the alternative homesteads were in the same geographical area with similar climatic and environmental characteristics.

Most of the respondents who reported history of malaria attack also reported that they spent much of their night time outdoors during the peak biting period and therefore could have picked the infection during that period when they are not yet sleeping under a mosquito net. The early biting activity of* An. arabiensis* could also expose individuals in the community who may not necessarily be involved in late outdoor activities.* An. arabiensis* has been shown to have early biting activities, which peak between 1900 hrs and 2000 hrs, with over 70% of biting activity occurring before 2200 hrs [26]. Therefore outdoor human nocturnal behaviour plays an important role in malaria transmission. The documented peak biting period coincided very well with the time that most of the individuals in Tubu village would be involved in late outdoor activities since the vector mosquito is also known to bite humans both outdoors and indoors [27].

*An. arabiensis *is an opportunistic species that feeds preferentially on humans in many parts of Africa but can be diverted to domestic animals as their density increases [28]. In Tubu village the presence and number of livestock near or within 3000 m from the homesteads could have some influence on the degree of malaria transmission at community and household levels. It is most likely that the risk of getting mosquito bites could have been reduced due to zooprophylaxis [29] especially in situations where the species predominantly displays zoophagic foraging tendencies. However, the benefit of keeping livestock close to human dwellings has been refuted by many authors from studies conducted in the Gambia [15] and the Ethiopian Highlands [9]. It is clear that the association between the presence of livestock as a risk factor and malaria transmission is a complex issue that needs further investigations in Botswana, where the ratio of cattle to humans is very high.

More than half of the respondents had travelled outside Tubu village in the last 8 months, to other more intense malaria endemic areas. Although active screening for malaria parasites was not done among the respondents, the responses to questions crafted to determine malaria risk implications of travel outside the study area indicated that travel to more intense malaria transmission areas exposed individuals to malaria. Reported visitors from malaria endemic areas could also have played an important role in importing malaria parasites to the village. In a study conducted in urban Kisumu, Kenya [14], children who reported spending at least one night per month in a rural area endemic to malaria were found to be at risk of contracting infections, indicating that exposure during rural travel was an important element of risk. The risks of acquiring malaria through travel were also observed in an urban area situated in the largest port of the Pacific Coast in Columbia [30].

Insecticide treated nets (ITNs) have become quite significant as the most practical method of mosquito control by protecting at-risk individuals from mosquito bites and hence malaria infections [31]. In this study participation of different organisations such as the council, clinic (Ministry of Health), and shops in supplying mosquito nets and the promotion done by the government could have resulted in overwhelming possession and use of mosquito nets by the community. Similar findings were reported in Nouma, Burkina Faso [22], and also in an urban area of Burkina Faso [32] where it was attributed to regional promotion. Reported mosquito net use in Tubu village was very high regardless of whether it is treated or not. The use of untreated mosquito nets has also been found to have some protective measures against mosquito bites [19, 33]. In Burkina Faso mosquito net use was relatively more frequent in summer than winter, thereby conferring protection from mosquito bites during the peak malaria transmission period [22, 32]. For an average family size of 6.8, with some homesteads possessing as many as 6 nets, it is most likely that each individual in the community had the opportunity to use a mosquito net. However, actual mosquito net use was not investigated and overreporting by the respondents was possible since ownership does not necessarily translate to actual use [34]. Innovativeness by the community in “mosquito net” making using waste maize-meal sacks has also been reported by the Doane College which implemented the Doane Nets Project in Kenya whereby recycled plastic bags were converted into mosquito nets. Since the quality of material used to make maize-bag “mosquito nets” was of major concern there is need for the Ministry of Health to promote the initiative and provide the community with the proper netting material for making more comfortable mosquito nets. This may however have intellectual property rights implications. Notwithstanding this, it shows that the community was aware of the protection given by nets and willing to contribute towards this initiative, thereby rendering the nets intervention more sustainable and not just waiting for handouts.

IRS, in addition to ITNs, is a mainstay of all national malaria control programmes in southern Africa. IRS is based on the assumption that mosquitoes feed and rest indoors, thereby coming in contact with the sprayed surfaces. Mosquito survey conducted in Tubu village showed that almost half of the vector mosquitoes caught resting indoors were blood-fed, an indication that the community was at risk of potentially infective mosquito bites. Therefore, more should be done in terms of indoor residual spraying, to deter mosquitoes from seeking indoor shelter. In addition the Ministry of Health should devise strategies to cover those high risk areas for malaria transmission that are considered inaccessible due to flooding. Although blood-meal analysis was not done, there is a high likelihood that some of the malaria vector mosquitoes might have fed from human beings, thus making the community vulnerable to potentially infective mosquitoes.

All households in Tubu village were within walking distance to the nearest health facility. Service at the health facility was reported to be satisfactory. This facilitated prompt treatment of malaria and other diseases. From similar observations made in Ghana it was suggested that in such situations a higher health education standard could have an additional effect in reducing malaria disease [8].

Most of the homesteads were surrounded by low vegetation cover not ideal as resting places for mosquitoes. In general, the area does not support thick vegetation and probably this resulted in daytime resting mosquitoes taking shelter in animal burrow pits. Incidentally, during the PRA discussions, the community lamented increased activities of pests such as squirrels and porcupine which create burrow pits that have become convenient resting places for mosquitoes. The location of homesteads relative to mosquito breeding habitats was one of the major risk factors for malaria transmission. All the study homesteads were sited within 3000 m from* An. arabiensis* infested water bodies, a distance considered to be within the general flight range of most mosquito species [35]. Such closeness of homesteads from breeding sites, in addition to the presence of animal burrow pits within the village, made the human population vulnerable to mosquito bites. Year-round sampling through the major BEP project established that larval breeding in Tubu village took place throughout the year in a variety of habitats created interchangeably by either rainfall or floods (BEP Fifth Report, 2012), thus exposing the community to year-round mosquito bites.

The presence of Culicine mosquitoes breeding in the same habitats exacerbated the problem of mosquito biting, though the species were of no medical importance in Botswana. Abundance of larvae and larval habitats and proximity of humans to larval habitats have been found to correlate very well with vector mosquito biting rates [11]. Close proximity of homesteads to vector mosquito breeding sites increased the risk of malaria in many countries including Uganda [10] and Ethiopia [12].

## 5. Limitations of the Study

The Ministry of Health changed its policy on malaria case management and recording in Botswana. As from October 2010 to date all suspected malaria cases are not handled at any clinic, Tubu included, but promptly referred to Gumare District Hospital. There is no record of malaria patients kept at the clinic of origin. All recordings are done at central/district hospital. Retrieving information from the district attendance register of patients originating from Tubu village was not possible because cases were pooled together onto one district register, with other patients presenting with other diseases. Furthermore the outcome on malaria patients referred to Gumare District Hospital is never conveyed back to the clinic of origin. As a result of these anomalies, individual malaria case history was obtained through interviewing the respondents. There is a possibility of both over- or underreporting of malaria episodes by the respondents. However a previous study among the same community/individuals showed that the community was well aware of the disease (95.6%) in terms of signs and symptoms (88.7%) and prevention measures (98.6%) and 100% of the respondents who had suspected a malaria attack sought medical treatment at the clinic [36]. Hence, our reliance is on the respondents' views on malaria history.

The study was conducted among 71 respondents representing 14.7% of the entire village population. Because of the small sample and population sizes of the study area, the results can only be extrapolated to other villages with similar demographic, ecological, and environmental characteristics and cannot be generalized to all malaria prone villages in Botswana. Similar case studies in different malaria prone settings in Botswana should be undertaken for comparative purposes.

## 6. Conclusions

In conclusion, socioeconomic status, exposure to mosquito bites through individual nocturnal outdoor activities, and mobility of the community were identified as high risk factors for malaria transmission in Tubu village. Limited use of malaria protective measures such as insecticide treated nets, house structure (traditional or modern), and close location of homesteads in relation to breeding sites exposed individuals to mosquito bites. However the number of mosquito nets possessed in each household and the farming status were not considered as risk factors for malaria. Hut/house eaves used as bedrooms by the respondents did not have any influence on the level, time, and frequency of mosquito bites. The overwhelming use of mosquito nets treated, untreated, or home-made proved to be a viable tool for malaria control in the village. Satisfactory service provided at and the close proximity of the health facility were added advantages to the community. Furthermore the community benefited from the low vegetation cover surrounding their homesteads, which did not provide enough shelter for even nuisance mosquitoes.

It is recommended that the Ministry of Health should develop a health education package encompassing some of the risk factors identified in this study and other indicated similar studies to be conducted in different settings. From our findings the most important issues to be addressed through the health education package include behavioural changes during nocturnal outdoor activities, ways of protecting oneself during visits to other malaria endemic areas, advice on the ideal location of homesteads in relation to mosquito sources, and promoting the use of insecticide treated eave curtains. Ways of promoting household based control methods, based on these specific risk factors, should be devised. The Ministry of Health should support community initiatives in mosquito net making, in addition to strengthening IRS activities in inaccessible flood prone areas of the Delta. Future research should comprehensively look at the role of other potential risk factors, such as zooprophylaxis and firewood smoke, in malaria transmission and on the possibility of incorporating the benefits derived, if any, into community based malaria control initiatives.

## Figures and Tables

**Table 1:** Sociodemographic characteristics of the respondents in Tubu village.

Characteristics	*n *	%
Sex		
Male	21	29.6
Female	50	70.4
Age		
18–29	23	32.4
30–39	18	25.4
40–49	7	9.9
50–59	10	14.1
60 and above	13	18.3
Marital status		
Single	36	50.7
Cohabiting	17	23.9
Married	10	14.1
Widowed	4	5.6
Never married	4	5.6
Educational status		
Never went to school	24	33.8
Primary	8	11.3
Junior secondary	31	43.7
Senior secondary	4	5.6
Tertiary	4	5.6
Ethnicity		
WaYei	64	90.1
HamMbukushu	3	4.2
Banderu/Herero	3	4.2
Farming practice		
*Molapo* farmers	60	84.5
Dry land farmers	1	1.4
Non farmers	10	14.1
Household status		
Household head	30	42.3
Household head's children	19	26.8
Household head's partner	15	21.1
Other relations	7	9.8

**Table 2:** Associations between various risk factors for malaria transmission in Tubu village determined by the Fischer and Pearson *χ* ^2^ test.

Risk factor	*P* value
Malaria episode versus period outdoors	0.051*
Household income versus malaria episode	0.018**
Malaria episode versus farming status	0.391
Malaria episode versus distance from water body	0.036**
Eave size versus mosquito bites	0.887^+^
Eave size versus frequency of mosquito bites	0.900
Eave size versus time of mosquito bites	0.746
Malaria episode versus number of nets possessed	0.496

**Significant association.

*Borderline association.

^+^Obtained from Fischer's exact test; the rest were obtained from Pearson's *χ* ^2^ test.

**Table 3:** Association between history of malaria episode and household/individual risk factors in Tubu village.

Risk factor	Odds ratio (OR)	95% confidence interval (CI)
Residential structure used as bedroom	11.781	3.868–35.885
Late outdoor activities	7.016	1.796–27.559
Travel outside Tubu village	2.700	1.004–7.260
Possession of mosquito nets	0.892	0.797–0.998

**Table 4:** Number of cattle owned by each respondent and distance of cattle kraals from homesteads in Tubu village.

Variable	*n *	%
Number of cattle		
None	20	28.2
<10	23	32.4
10–<50	20	28.2
50–100	6	8.5
>100	2	2.8
Distance of kraal from homestead		
<3000 m	2	4.0
>3000 m	49	96.0
